# Recognizing the Importance of Design, Content, and Delivery Features of Health Animations for Preventive Health Behaviors: Realist Review

**DOI:** 10.2196/79769

**Published:** 2026-04-23

**Authors:** Kathleen McCorry, Gregory Maniatopoulos, Linda Errington, Ellie Land, Michael Craig, Santosh Vijaykumar, Till Bärnighausen, Nicola O’Brien

**Affiliations:** 1 Heidelberg Institute of Global Health, Faculty of Medicine and University Hospital, Heidelberg University, Heidelberg, Germany; 2 School of Business, University of Leicester, Leicester, United Kingdom; 3 Walton Library, Newcastle University, Newcastle upon Tyne, United Kingdom; 4 School of Computing, Engineering and Digital Technologies, Teesside University, Middlesbrough, United Kingdom; 5 School of Psychology, Faculty of Health and Wellbeing, Northumbria University, Northumberland Building, Newcastle upon Tyne, NE1 8ST, United Kingdom, 44 01912274158; 6 Department of Global Health and Population, Harvard TH Chan School of Public Health, Harvard University, Boston, MA, United States; 7 Africa Health Research Institute, Somkhele and Durban, South Africa

**Keywords:** animation, health behavior, health communication, preventive medicine, realist review

## Abstract

**Background:**

Animated messages designed to promote preventive health behaviors (health animations) are a prevalent form of digital health communication globally and are used across a variety of health behaviors. Health animations are visual and can be less reliant on language, helping to reduce health literacy barriers and inequities. Being easily and inexpensively shared, they are a potentially powerful tool for disease prevention. Evidence suggests that animations can be effective in health and non–health care settings. Despite a plethora of health animations in existence and their potential reach and scale, evidence underpinning their design and application, including the potential causal mechanisms at work, is often limited or unknown.

**Objective:**

This realist review aimed to understand why, how, for whom, to what extent, and when health animations designed to promote preventive health behaviors work.

**Methods:**

The review was conducted in accordance with the Realist and Meta-narrative Evidence Syntheses: Evolving Standards (RAMESES). Peer-reviewed publications identified through database searching from inception until April 27, 2025, as well as gray literature, were considered for inclusion. Animations designed to promote preventive health behaviors in any population and evaluations using any design were included. Animations that could not be viewed, were designed to treat illness or disease, or were part of multicomponent interventions were excluded. Data were appraised for their relevance, rigor, and richness. Data syntheses sought to produce context-mechanism-outcome configurations, contributing to a program theory of health animations. International stakeholder workshops with professionals and members of the public were used to sense check findings and refine the program theory.

**Results:**

This review synthesized data from evaluations of 48 health animations. Within the data, design, content, and delivery constructs were identified, including audience or challenge representation, using storytelling, evoking emotion, accessibility, and other contexts. These contexts enabled the triggering of key mechanisms, such as identification, transportation into a story, attention, building self-efficacy, and cognitive processes. The evidence available for synthesis in the building of our program theory of health animations was limited by a lack of data on behavioral outcomes, meaning that the theory is largely derived from evidence of the contexts and mechanisms influencing key determinants known to affect behavior. These key determinants include behavioral intentions, skills and attitudes, and knowledge as a key factor in raising awareness of the need to change behavior.

**Conclusions:**

This realist review advances the understanding of the impact of health animations designed to promote preventive health behaviors by providing insight into the design, content, and delivery features at work. Our program theory describes the specific contexts and mechanisms that influence evidence-based determinants of behavior and behavior change. These contexts and mechanisms, therefore, should be considered during health animation design and development processes. A set of 10 recommendations is provided to this end.

## Introduction

### Background

Effective public health communication plays a crucial role in empowering individuals with information and skills to have more control over the decisions and behaviors that impact their health. Modifiable behavioral risk factors, such as sedentary behavior, unhealthy diets, not attending health screenings, and high-risk sexual behaviors, contribute to between a quarter and a half of the global burden on health and social care, as well as deaths [1-3]. Health communication science can critically inform the strategies required to ensure that accurate and accessible information and support are provided to individuals, communities, and populations to promote health and positive health behaviors, and reduce subsequent morbidity and mortality [4].

Effective health communication strategies need to address health literacy, defined as the knowledge and competencies needed to access, understand, and use health information [5]. Including visual stimuli within health communication is encouraged, particularly when trying to reach individuals disadvantaged by lower literacy and education levels; knowledge, understanding, attention, and recall are improved when visual images are used in health communication compared with using text alone [6]. This is supported by the picture superiority effect from cognitive psychology, which shows that visual information is more memorable than textual information. This suggests that visual information is more effectively encoded into memory, as it activates both verbal and visual memory systems, strengthening memory formation and subsequent recall [7].

Health animations, defined as simulated motion pictures depicting the movement of drawn (or simulated) objects [8] related to health, are a highly prevalent approach to visual health communication. Due to their digital nature, animations can be easily and inexpensively shared via the internet, apps, and social media, catalyzing their widespread global application as a digital health solution [9] to deliver public health information, promote health behavior change, and prevent ill health. Compared with paper-based or in-person health communication, animations have greater reach and scale, overcoming geographical barriers and exploiting the rapid rise and evolution of social media platforms (eg, TikTok, WhatsApp, and Instagram) and online mediums (ie, local and international governmental, nongovernmental, voluntary sector, charity organizations, or websites).

Emerging reviews and meta-analytical evidence of animations suggest that there are benefits to learning and cognitive load from animations as educational tools for patients [10,11], health care professionals [12], and in non–health care settings [13]. However, evidence of health animations in the promotion of public health preventive behaviors, such as lifestyle, hygiene, and preventive health appointment uptake—which can prevent and control communicable and noncommunicable diseases—remains unclear. To date, evidence relies on individual empirical studies [14-16] investigating the impact of health animations on behaviors and social cognitive determinants (eg, recall, knowledge, and intention). These individual studies exhibit substantial heterogeneity in study design, comparators, target health behaviors, target audiences, and contextual complexities in the animation design and content. Therefore, rather than attempting to synthesize evidence for the effectiveness of health animations promoting preventive health behaviors from a pool of heterogeneous studies, we adopt a realist review approach to iteratively identify and synthesize evidence to uncover the specific causal mechanisms being triggered by animations that, in turn, facilitate certain outcomes.

### Aim of the Review

The aim of this realist review was to identify and synthesize evidence about health animations to understand how, why, for whom, to what extent, and in which contexts they are expected to produce their effects. We aimed to generate knowledge about the causes of outcomes in particular circumstances in terms of the context-mechanism-outcome configurations (CMOCs) of realist reviews and syntheses. By understanding how and why health animations and the processes underpinning them work (or fail to work), we aimed to translate key learnings into a set of recommendations to inform the design, development, and implementation of future public health animations.

## Methods

### Overview

This realist review and synthesis was conducted in accordance with the Realist and Meta-Narrative Evidence Syntheses: Evolving Standards (RAMESES) [17-19] and a published protocol [20]. Textbox 1 provides definitions of key realist concepts. The realist review methodology and structure are well-suited to the complexity and heterogeneity of health animations and the methods used to evaluate them. Health animations are used across a variety of health behaviors and therefore can produce multiple varied outcomes. While conventional systematic reviews focus on outcomes, realist reviews focus on the contexts and the mechanisms they trigger to produce those outcomes. Furthermore, realist methodology does not seek to separate and categorize the elements of the evidence being explored but instead finds causation and configurations that lead to a better understanding of how and why effects are produced [21]. This is achieved by identifying contexts and mechanisms and examining how they interact to produce outcomes; once identified, CMOCs can be developed. The following 5 iterative steps were undertaken with some elements within the steps occurring simultaneously.

Textbox 1. **Definitions of key realist concepts [19,21].**

**Context**

- A set of factors or conditions influencing how an animation operates or works
- Context triggers mechanisms

**Mechanism**

- Hidden, underlying, context sensitive, causal force triggered by context
- Mechanisms are behind “why” and “what” makes things happen
- They are transferable
- Can be internal cognitive or psychological processes (ie, developing understanding and the building of confidence)
- Not states of being but processes

**Outcome**

- Outcomes vary including intentions, behaviors, and attitudes
- Outcomes help make what is functioning as context clearer

**Context-mechanism-outcome configuration (CMOC)**

- A lens through which to think about how things are working
- Support the knowledge being generated through the synthesis
- Help to tell the story of what is happening

**Program theory**

- Underpinned by theory (explicitly or not) and reflecting what is understood about causation
- Seeks to explain how a program (ie, an intervention) is expected to work
- May include multiple CMOCs and can be expressed in diagram or narrative format

**Demiregularity**

- Semipredictable behavioral patterns reflecting individuals’ thoughts and actions that are influenced by contextual differences

### Step 1: Defining the Review Scope and Identifying Existing Theories

Theories and models across multiple disciplines were explored for insights and understanding of what may influence how health animations are perceived, interpreted, or experienced. These insights were discussed within the review team to develop initial program theories. Exploratory background searches were conducted to identify literature on the use of health animations and their impacts and effects, helping to define the review’s scope.

### Step 2: Search for Evidence

Initial searches of electronic databases were conducted from database inception until July 10, 2023, with ongoing searching throughout the review process (in accordance with the iterative nature of realist reviews). A final search of electronic databases was conducted on April 27, 2025. The databases searched were MEDLINE, Scopus, CINAHL, EMBASE, PsycINFO, ProQuest Social Science, Web of Science, and the Cochrane Library. The search strategy, combining relevant Medical Subject Headings (MeSH) terms and keywords for concepts of animation, health behavior, and behavior change, is available in Multimedia Appendix 1. Reference lists were checked, and websites of governmental, nongovernmental, and third-sector organizations known to promote public health were searched to identify additional health animations. Google and YouTube were also searched using keywords to identify additional health animations and organizational sources of animations.

Rayyan (Rayyan Systems, Inc) was used to manage the records, including deduplication. Title and abstract screening was conducted by 1 reviewer (KMC), with a random 10% sample screened independently by another reviewer (NO’B). Full-text screening was conducted independently by 2 reviewers (KMC and NO’B). The authors were contacted to provide animations when they were not publicly available. Textbox 2 presents the inclusion and exclusion criteria.

Textbox 2. **Inclusion and exclusion criteria of publications**

**Inclusion criteria**

- Participants: any
- Interventions: animations communicating health messages designed to promote preventive health behaviors or social cognitive determinants thereof
- Comparators: other interventions, usual care, no intervention, or no comparator
- Types of study designs: any
- Contexts: any
- Outcomes: any, including, but not limited to: health behavior, behavioral cognitions (attitude, intention, and self-efficacy), knowledge, awareness, emotions, memory, and recall.

**Exclusion criteria**

- Animations designed to treat illness and disease.
- Multicomponent interventions where animation effects could not be isolated from other components.
- No access to animation

### Step 3: Selection and Appraisal of Evidence

Evidence was selected using the criteria outlined in step 2 of the protocol [20]. Peer-reviewed and gray literature publications were included where they could potentially contribute to building the program theory. The relevance, rigor, and richness of the data were used to assess the quality of evidence [22]. Questions asked of the data in relation to relevance included: Does the evidence help develop CMOCs and contribute to their advancement? Is the building and testing of program theory supported by this evidence? Does it speak to the research question? Questions asked in relation to richness included: Is there a sufficient level of description about the development of the intervention and the theories and concepts underpinning it? Was there discussion about factors that impacted the functioning or outcomes of the intervention? Were details provided about how the intervention was meant to work? Was this information general or specific to the intervention? Questions asked in relation to rigor included: What methods were used to generate the data? What are the credibility and trustworthiness of these methods?

### Step 4: Data Extraction

Data extraction was conducted by 1 reviewer (KMC) and checked by a second reviewer (NO’B). Bespoke data extraction sheets were developed to record study and animation characteristics. Study characteristics included information about participants, outcomes, findings, health behaviors targeted, and theories influencing animation design. Animation characteristics included length, use of sound, color, language, and text, development information, composition, character profiles, use of cultural indicators, symbols, and metaphors.

### Step 5: Data Synthesis and Theory Refinement

Data synthesis and theory refinement were developed through multiple viewings of the animations, discussions within the review team, and consideration of the identified theories and models. The initial synthesis and CMOC development involved KMC, NO’B, and GM reviewing the data to identify patterns (ie, demiregularities) and elements that the animations shared to define the “if” (ie, contexts) and to list the “then” (ie, outcomes). Data were then interrogated to define the “because” (ie, mechanisms), resulting in if-then-because (context-outcome-mechanism) statements [23]. The integrity of the if-then-because statements was questioned as discrete statements by considering the included data and their potential interpretations in relation to the initial program theories identified in step 1. In doing so, an emerging program theory was developed.

Four stakeholder workshops were held to sense check preliminary findings and to discuss and refine the if-then-because statements and the emerging program theory. Two international online workshops were conducted with professionals in April 2024 (n=21), allowing for the testing and refinement of the program theory with individuals who develop, commission, and/or promote animations or who work with individuals or systems that may benefit from health animations. Two in-person workshops were held with members of the public in the United Kingdom in May 2024 and June 2024 (n=18), enabling the testing and refinement of the program theory with public stakeholders targeted by and exposed to health animations. Following these workshops, feedback was used to review the if-then-because statements and translate them into CMOCs. The CMOCs were tested against the data and refined as required. They were then used to develop a set of recommendations to inform the design, development, and implementation of future public health animations. Both the CMOCs and the recommendations were presented to professional and public stakeholders for feedback during a final online workshop in September 2024 (n=17).

### Ethical Considerations

Ethical approval for the stakeholder workshops was granted by the Northumbria University Research Ethics Committee (reference #6803). Stakeholders provided informed consent before the workshops, and their privacy and confidentiality were ensured. Public stakeholders were remunerated for their time and contribution with a £25 (US $33.8) shopping voucher.

## Results

### Overview

**Figure 1.**
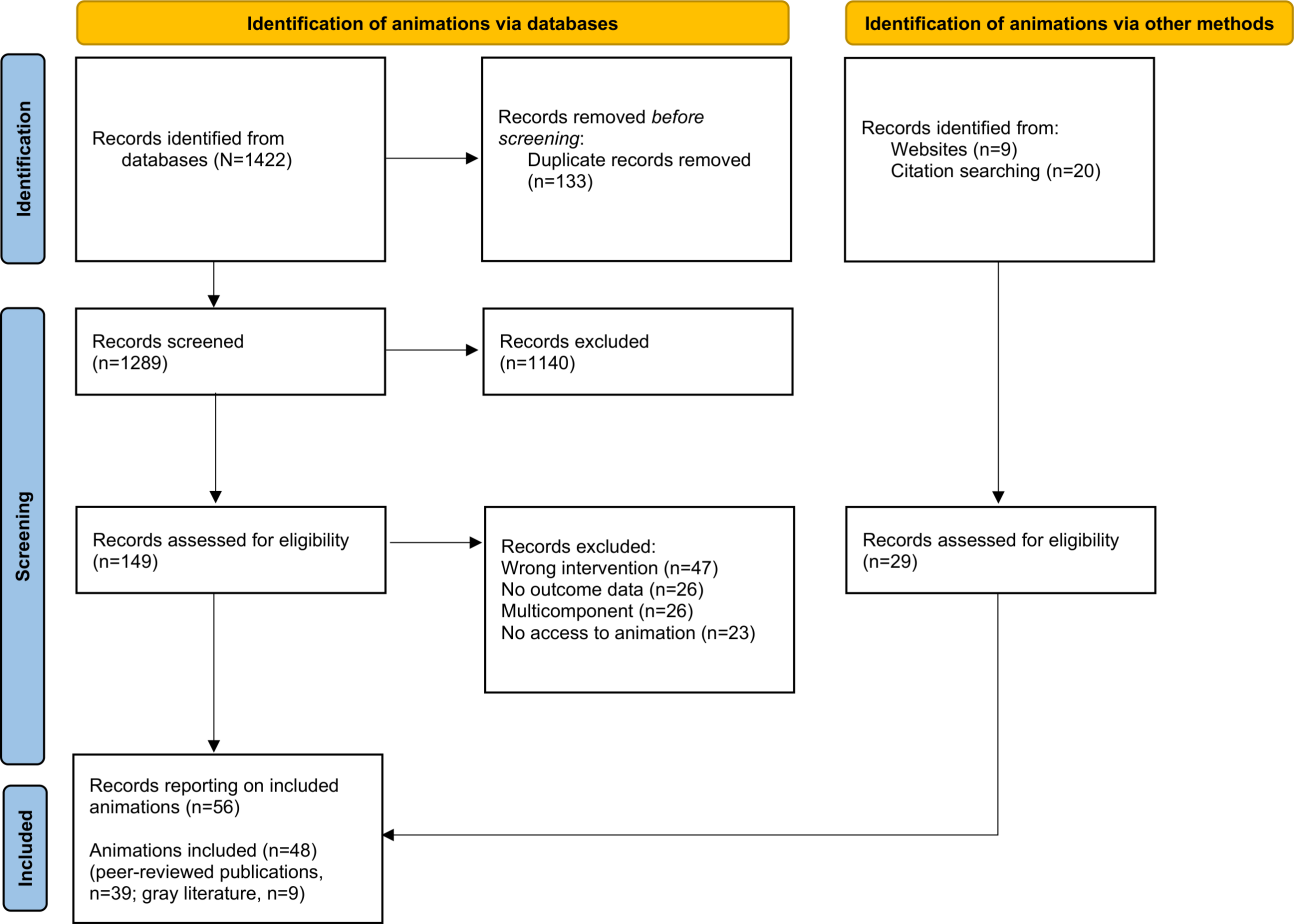
Flow diagram of animations included in the realist review, adapted from PRISMA (Preferred Reporting Items for Systematic Reviews and Meta-Analyses).

The final sample for this review included 48 unique single animations or series of animations; 39 were reported in 47 peer-reviewed publications [14-16,24-67] and the remaining 9 were gray literature [68-76]. Figure 1 displays the flow of the selection of animations. Categories of health behaviors targeted were communicable diseases and hygiene behaviors (n=11), vaccination (n=10), general health promotion (n=8), screening (n=5), substance use (n=5), sexual health (n=4), nutrition (n=3), and oral health (n=2). Study designs included: randomized controlled trials (n=18), randomized studies (n=12), pre-post studies (n=8), mixed methods studies (n=5), and cross-sectional studies (n=4).

Of the 48 animations included, 28 were single animations and 20 were animation series; for the purposes of data synthesis, animation series were considered as single animations. Outcome measures included knowledge, behavioral intentions, attitudes, skills, acceptability, and viewer engagement; 8 studies also included behavioral outcomes. Many animations reported details of co-design processes, for example, in a study by Willis et al [65], and theories and models (eg, social cognitive theory [47], elaboration likelihood model [24], and theory of planned behavior [50]) that informed animation development, evaluation, and outcome measures, and provided key insight and information in the building of the program theory.

Most animations used storytelling to convey messages (n=22), while others used entertainment elements (n=18), storytelling elements (n=4), or no storytelling/entertainment elements (n=4). Textbox 3 provides an explanation of the different categories of entertainment that were used. Further study design details, including the demographic and geographical target audience of the animations, as well as the animation characteristics, such as the use of sound, language, text, and length, are presented in MultimediaAppendices 2  3 [14-16,24-76].

Textbox 3. **Explanation of entertainment categories used in animations**

**Storytelling**

- Characters, plot line, beginning/middle/end, and entertainment elements

**Storytelling elements**

- Testimonials, persona building, informational plus persona/character/situation building

**Entertainment elements**

- Anthropomorphism, characters in action, and so on

**No storytelling or entertainment elements**

- Images only function to inform

### Realist Analysis

**Figure 2.**
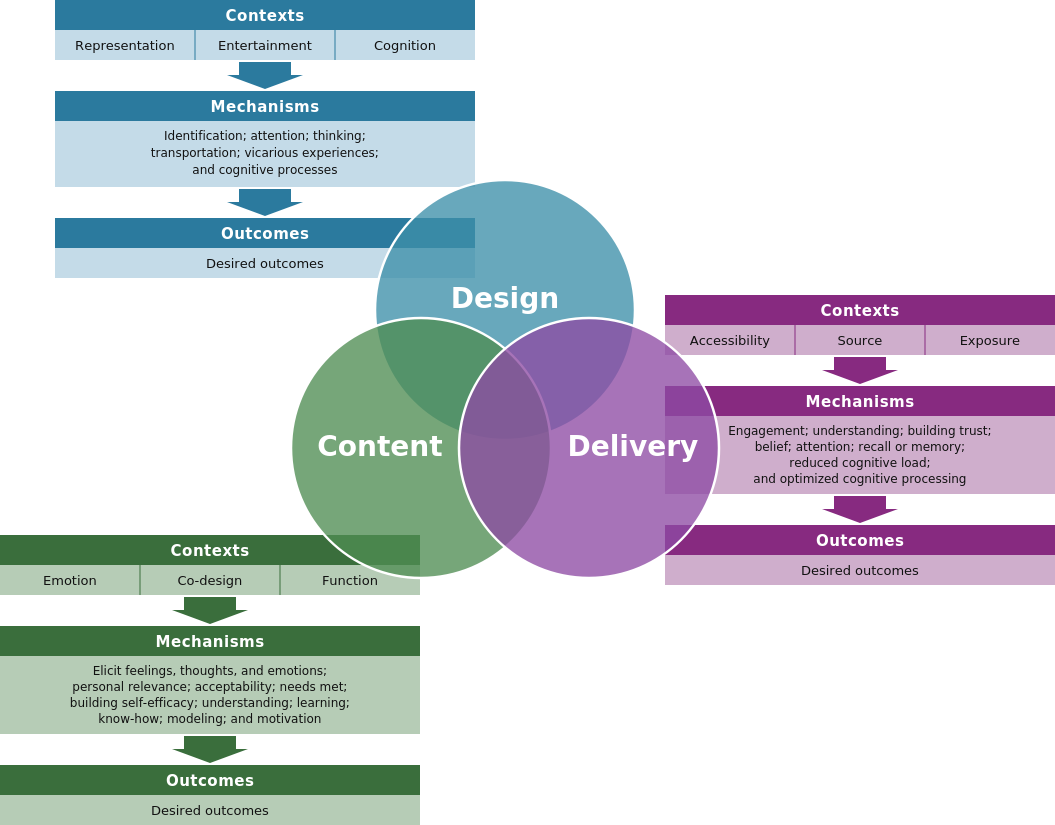
Diagram depicting high-level constructs of animation design, content, and delivery, as well as the related contexts, mechanisms, and outcomes identified in the analysis.

The main characteristics contributing to understanding why, how, for whom, to what extent, and in which contexts health animations are expected to promote preventive health behaviors were considered within 3 high-level constructs: design, content, and delivery. These constructs did not operate in isolation but often worked together to enhance effects. It is within these constructs that the contexts, mechanisms, and outcomes identified in the analysis were situated (Figure 2). Consequently, the following 9 CMOCs were developed and tested with stakeholders (Table 1). The final column of Tables S1 and S2 in MultimediaAppendices 2  3, respectively, details which CMOCs were associated with each animation included. Additional detail on the constructs, contexts, and mechanisms is provided in Multimedia Appendix 4.

**Table 1.** Context-mechanism-outcome configuration (CMOC) descriptors and the number of animations included in the review shaping and relating to each CMOC.

CMOC	Descriptor	Number of animations
Design CMOC 1: representation	If the animation is designed to represent a particular audience (or shared challenge), desired outcomes are more likely to be achieved, because the audience being represented identified with its elements allowing them to pay greater attention and think about what they are watching.	27
Design CMOC 2: entertainment	If the animation uses storytelling, desired outcomes are more likely to be achieved, because the audience will be transported into the story.	44
Design CMOC 3: cognition	If the animation is designed to facilitate efficient cognitive processing, then the desired outcomes are more likely to be achieved because cognitive mechanisms were enhanced.	44
Content CMOC 1: emotion	If the animation presents its content in an evocative manner, then desired outcomes are more likely to be achieved, because the animation content elicited powerful feelings, thoughts, and emotions.	22
Content CMOC 2: co-design	If the content of the animation is co-designed, then desired outcomes are more likely to be achieved, because the animation is more relevant, acceptable, and meets the needs of the target audience.	14
Content CMOC 3: function	If the content of the animation presents the challenge and the solution, then desired outcomes are more likely to be achieved, because the content of the animation increased audience understanding and provided ‘know-how’ information that helped the audience feel empowered and believe in their capacity to deal with the challenge.	44
Delivery CMOC 1: accessibility	If the animation is delivered in an accessible manner for the target audience, then desired outcomes are more likely to be achieved, because they will be better able to understand and engage with the information.	43
Delivery CMOC 2: source	If the animation is delivered by a source that is perceived to be credible, reliable, and trusted, then desired outcomes are more likely to be achieved, because they believed and engaged with the information and therefore better attended to and recalled it.	33
Delivery CMOC 3: exposure	If the mode, channel, and/or intensity of the delivery of the animation encapsulates the purpose and needs of the target audience and/or behavior, then desired outcomes are more likely to be achieved, because the potential exposure to the animation optimizes cognitive processing and responds to the needs of the audience.	31

### Design

The construct *design* refers to the ways in which health animations are constructed to express the ideas and messages they are trying to communicate. How an animation looks, feels, and sounds, as well as its length, use of symbols and metaphors, character profiles, and other features, are all products of the design.

#### Design CMOC 1: Representation

Twenty-seven animations (reported in 27 publications [16,24,25,32,36,37,40,41,43-45,47,49,54,56-58,62,65,68-74,76]) were identified as shaping and relating to this CMOC. Within the context of designing animations to represent audiences or specific challenges, the mechanism of identification emerged as contributing to outcomes. Once the audience identifies themselves or the challenge depicted in the animation, the mechanisms of attention and thinking may also be triggered. Professional and public stakeholders felt that this finding resonated with their experiences, represented their understanding, and promoted inclusivity. Professional stakeholders further commented that this CMOC reduces unconscious biases and that identifying oneself is key to engagement, increasing accessibility, particularly for marginalized communities. Some stakeholders expressed that the desired outcomes and target audience should guide how representation is used.

An example of this CMOC in practice can be seen in the animated series “Meena” [73]. This animation was purposely designed to reflect the lives of girls in South Asia, with special attention paid to accurately representing the look and sound of characters, their environments, and their typical experiences.

#### Design CMOC 2: Entertainment

Forty-four animations (reported in 52 publications [15,16,24-33,35-45,47,49-76]) were identified as shaping and relating to this CMOC. Storytelling and entertainment include the use of characters, plot, storylines, imagery, visuals, and other elements. Additionally, anthropomorphism was used to make biological processes easier to understand and entertaining at the same time, such as representing white blood cells as soldiers [35]. Transportation is a mechanism that refers to one engaging in a way that enables greater attention and creates vicarious experiences for the viewer as they become caught up and entertained by the story. Stakeholders supported this CMOC expressing that storytelling or entertainment make messages easier to remember and more relatable. Professional stakeholders expressed that storytelling/entertainment can be facilitated without reliance on language, increasing accessibility for those with language and/or literacy barriers.

*The Story of Cholera* [69] is an example of an animation that fully uses storytelling. This animation tells the story of a young boy whose father contracts cholera and becomes very ill. The boy subsequently learns how to prevent cholera and teaches the people in his village hygiene practices to prevent the disease. The storytelling in this animation is supported by the use of color, music, sound effects, and narration. The entertainment context often works in conjunction with other contexts such as emotion. An example of this can be seen in an animation depicting the story of a pregnant mother’s decision-making journey as she learns about the flu vaccine and considers the health implications (and experiences the associated emotions) for herself and her baby [57].

#### Design CMOC 3: Cognition

Forty-four animations (reported in 52 publications [14-16,24-52,54-61,63,64,66-73,75,76]) were identified as shaping and relating to this CMOC. Design features that facilitate efficient cognitive processing, such as the use of familiar characters and structures, cultural representations in terms of metaphors and symbols, composition of images, and other elements, can trigger and enhance cognitive mechanisms. These cognitive mechanisms may include memory or recall, thinking, learning, attention, and cognitive load. Stakeholders agreed with the CMOC while also cautioning against overwhelming audiences with the heavy use of text and multiple simultaneous messages.

An animated series for children, *Akili and Me*, which has been culturally adapted and was reported in 2 studies [32,47], contains many elements described in this CMOC. The studies discuss the use of characters and structures that children become familiar with as well as the use of color and music to ease cognitive load and improve learning, recall, and retention of information.

### Content

The construct *content* refers to the message itself, the information it contains, and how it is expressed.

#### Content CMOC 1: Emotion

Twenty-two animations (reported in 27 publications [16,24,25,27,28,30,39-43,49-51,53,57,58,63-65,67-71,74,75]) were identified as shaping and relating to this CMOC. This CMOC was met with much enthusiasm by stakeholders, with remarks that emotion has the power to increase message engagement and behavioral motivation. The value, relevance, and importance of evocative messaging were recognized by all stakeholders, with some cautioning that consideration for the emotions and feelings being elicited is needed so as not to induce or trigger distress or negative thoughts, emotions, or behaviors. Professional stakeholders communicated that evocative content could be powerful enough to be impactful without the use of language.

CoVideo is an animated video that evokes emotion through storytelling, character movements, the use of colors, sounds, and other elements [16]. This short animation is an example of how emotions can be expressed and elicited in an audience without using language.

#### Content CMOC 2: Co-Design

Fourteen animations (reported in 15 publications [24-26,33,37,43,45,56,57,59,60,65,68,73,76]) were identified as shaping and relating to this CMOC. When the target audience of an animation co-designs the content, the animation can reflect what is important and relevant to them, triggering the mechanisms of perceived personal relevance, acceptability, and the feeling that individuals’ needs are being met. Stakeholders agreed that co-design provides crucial insight into the lived experiences of the target audience, while reducing barriers to messages, increasing accessibility and identification, and thereby meeting the needs of the intended viewer.

Digital Animation in Service Improvement is an example of an animation intervention that engaged in co-design at multiple points in time and with different groups [43]. This animation was developed over a 6-month period in consultation with individuals who were representative of the target audience as well as relevant medical professionals. These groups informed the key messages, cultural appropriateness, and other elements of the design and content of the animation.

#### Content CMOC 3: Function

Forty-four animations (reported in 52 publications [14-16,24-30,32-39,41-45,47-64,66-76]) were identified as shaping and relating to this CMOC. Animations that present a challenge and its solution can trigger mechanisms such as building self-efficacy, understanding, and learning. Educating audiences about behavioral challenges is largely insufficient for behavior change but showing *how* to overcome these challenges can increase motivation by providing critical know-how information. When animations present this type of content, they often provide modeling and vicarious learning opportunities, increasing self-efficacy and motivation. Stakeholders agreed with this CMOC and felt that providing solutions is vital to empower individuals to overcome health challenges, and that solution-based content may complement and increase the effectiveness of emotion-based content.

An animation providing information about menopause serves as an example of this CMOC [44]. The short videos explain what menopause is, what symptoms may be experienced, multiple potential solutions for women experiencing symptoms, and resources they can use to find more information or get assistance.

### Delivery

The construct *delivery* refers to how health animations are experienced, including who is delivering the message, what platforms are being used, and how and through what modes animations are accessed. The delivery of health animations also includes the intensity of exposure.

#### Delivery CMOC 1: Accessibility

Forty-three animations (reported in 50 publications [14-16,24-39,41-67,69-71,73]) were identified as shaping and relating to this CMOC. This CMOC recognizes that accessibility is key to the delivery of health animations. When animations are delivered in an accessible way for the target audience, mechanisms of engagement and understanding are triggered. Accessible delivery can vary for different audiences and challenges (eg, in-person or online, viewing in a group, alone, or in the presence of a health care professional). Accessibility also relates to literacy levels and meeting audiences’ needs. Professional stakeholders felt that this CMOC addressed an important consideration and made sense within their organizations. The settings where animations are shown and how they are accessed were discussed by stakeholders, including the need to address barriers to comprehension and literacy for marginalized communities.

A series of GIFs promoting COVID-19 preventive behaviors display the multiple meanings of accessibility expressed in this CMOC [56]. First, the format—GIFs—are small image files that are easily shared on mobile devices and, second, the GIFs are free of written or spoken words (ie, languageless), thereby communicating the messages through visual storytelling only and addressing potential language and literacy barriers.

#### Delivery CMOC 2: Source

Thirty-three animations (reported in 41 publications [14-16,24-39,41-45,47,50,51,53-60,63-66,68,73]) were identified as shaping and relating to this CMOC. Source, in relation to this CMOC, can refer to the producer, person, or platform delivering the message, as well as the setting in which it is viewed. If the source is perceived to be credible, reliable, and trusted by the viewer, it can trigger mechanisms such as belief, engagement, and building trust, thereby enhancing attention, recall, and memory. Stakeholders felt that when a source is recognized and trusted, audiences engage more quickly and easily, and would be more likely to act on the message. Many stakeholders recognized that perceptions of credibility are influenced by online and offline misinformation and disinformation, and that the digital and nondigital settings where people are exposed to animations impact the perceived trustworthiness.

Many animations displayed clear information about the source of the information contained within them and who was providing the information. One example of this is an animation promoting flu vaccines for pregnant women, where there is clear information about the source of the animation and how or where to get additional information or have questions answered [57].

#### Delivery CMOC 3: Exposure

Thirty-one animations (reported in 38 publications [14-16,25-32,34-39,41,42,44,45,47,49-51,53-60,62-65,68]) were identified as shaping and relating to this CMOC. This CMOC refers to the way audiences are exposed to health animations in terms of auditory and/or visual channels, cognitive pathways, and intensity of viewing (single or series, over time or all at once, and so on). If these and other delivery elements meet the needs of the target audience, then cognitive load may be decreased, thereby further optimizing cognitive processing. Stakeholders expressed that the CMOC was clear and made sense to them and that addressing audiences’ needs can maximize an animation’s impact.

A series of short animations, titled “Respect Your Brain,” reported in 2 studies [36,37], is associated with this CMOC. These well-received short animations have a similar format throughout the series and were delivered online as a package.

These CMOCs were used to develop a set of 10 recommendations for the design, content, and delivery of future health animations in research and practice (Textbox 4).

Textbox 4. **Research and practice recommendations for the design, content and delivery of health animations**

- *Design* the animation to *represent the target audience* (or target behavior or challenge) so that the audience can identify with and pay greater attention to the animation elements
- *Design* the animation to be *entertaining and use storytelling* so that the audience can be transported into the story
- *Design* the animation to *facilitate efficient cognitive processing* (memory, recall, learning, and so on).
- *Content* should be presented in an *evocative manner* so that powerful feelings, thoughts, and emotions are elicited.
- *Content* should be *co-designed* so that the animation is more relevant, acceptable, and meets the needs of the target audience.
- *Content* should include *information and a solution* so that the audience understands how, and feels confident, to act on the animation’s information.
- *Content and delivery* should be *accessible* (language, literacy levels, free of jargon, and so on) to the target audience so that the messages can be understood.
- *Deliver* the animation in a *setting* that the target audience can *easily access* (online health or community center, public space, and so on) so that they can engage with and understand it.
- *Deliver* the animation using a *source* (individual or organization, platform, and/or setting) that is *perceived as credible* by the target audience so that they trust and engage with the message.
- *Deliver* the animation using a *mode* (in-person, digital, or both), *channel* (auditory or visual pathways), and *intensity* (single or series, one time or repeated) that *meet the needs* of the target audience so that cognitive load is reduced**.**

## Discussion

### Principal Findings

This realist review identified and synthesized evidence from 48 health animations providing an understanding of how, why, for whom, to what extent, and in which contexts health animations designed to promote preventive health behaviors work or fail to work. With input from public and professional stakeholders, we developed a program theory comprising high-level constructs and CMOCs, which were translated into a set of recommendations to inform the design, development and implementation of future public health animations.

The review produced 4 key findings. First, there are three interrelated high-level constructs (ie, design, content, and delivery) that often work synergistically. Furthermore, when exploring these constructs and their use in health animations, they were broken down into specific contexts thought to trigger causal mechanisms.

Second, entertainment was identified as a key context used in many health animations, through storytelling and its elements. Storytelling is based on the theory that humans understand and give meaning to their life experiences by telling stories [77]. Moyer-Guse et al [78] discussed the role of the cognitive mechanisms of persuasion and transportation by a story in relation to childhood vaccination attitudes and further discussed the power of narratives and entertainment to affect attitudes and beliefs. More broadly, “entertainment education” approaches have historically been used for health communication globally and to good effect across a range of health domains [79]. In this review, the terms “storytelling” and “entertainment” were used instead of “narrative” and “didactic,” as conversations with the research team and stakeholders revealed that the terms “narrative” and “didactic” had the capacity to take on multiple meanings and cause confusion.

Third, the entertainment context often operated in conjunction with other contexts identified in the review. For example, when emotionally evocative content was present, storytelling, and/or entertainment were also present. This finding is in line with evidence that stories can capture audiences’ attention, educate and entertain them, and evoke emotions [80]. Concurrent with evoking emotions, is the possibility of psychological reactance, which, in the context of health communication refers to an audience’s resistance to act upon a health message in the intended way due to a perceived threat to their freedom [81].

Fourth, contexts that facilitated accessibility were deemed particularly valuable to stakeholders, who frequently discussed the important challenge for health communications to address health literacy. Health literacy is a key social determinant of health [82]. Literature reviews consistently show that low levels of health literacy are associated with worse health outcomes, including higher mortality rates, more hospitalizations and emergency department visits, and poor engagement with healthy behaviors, health messages, and preventive health care services [83,84]. Educational attainment has been shown to be the most important determinant of health literacy, followed by other socioeconomic factors such as financial resources [85]. Therefore, within the current context of health animations, it is of paramount importance to consider the role of these factors in determining the impact of an animation. The contexts that facilitate accessibility to and comprehension of the information and resources provided in health animations were present across all three of the high-level constructs identified in this review and therefore equally require full consideration in efforts to enhance health literacy.

### Recommendations for Future Research and Practice

Based on the findings of this review, we suggest a set of recommendations for researchers and practitioners to consider when designing and developing future health animations. The relative weight or focus that can be given to each recommendation may depend on multiple factors including the health topic, audience, time, and financial resources available to develop the health animation. For example, when developing an animation designed for a global or multilingual audience, to maximize its potential reach without requiring additional resources to develop multiple versions in different languages, it would be of paramount importance that the animation does not rely on written or spoken language to be understood, thereby reducing language and literacy barriers. Instead, storytelling, entertainment, and emotional elements could be exploited to ensure that the animation visuals alone can work to promote the target health behaviors. It would also be important to consider the most acceptable and utilized modes for animation delivery (eg, social media, instant messaging, and screens in public places) that meet the differing needs of subgroups in the target population. Researchers and practitioners should also consult these recommendations when reviewing data from individual studies evaluating health animations to appraise the specific features of the animation design, content, and delivery.

### Limitations and Strengths

Few evaluations of animations provided data on behavioral outcomes and therefore, our program theory largely reflects the contexts and mechanisms at play when producing effects on key evidence-based behavioral determinants. The behavioral determinants that the animations were found to impact included evidence-based correlates of subsequent health behavior, namely behavioral skills, attitudes, and intentions [86]. Critically, interventions targeting behavioral skills and attitudes are known to produce small effects on health behavior [87], and therefore animations we identified as evidencing impact on these determinants may also yield positive behavioral changes. Moreover, many of the animations identified in our review reported knowledge gains, and even though interventions targeting knowledge are known to produce only minimal effects on behavior [87], knowledge often plays a critical role in health behavior change in terms of raising awareness of the health problem or topic, the need to change behavior, and its link to behavioral and self-regulation skills [88,89].

Future studies are urged to collect both short- and long-term behavioral data when evaluating health animations to expand the evidence base regarding their effect on behavioral outcomes. Furthermore, future research is needed to investigate the role animations could play as part of wider behavior change interventions, potentially targeting not only the more impactful individual determinants of behavior but also sociostructural factors [86,87]. Finally, investigations into potential dose-response effects through repeated exposure to an animation (or a series) are required to further our knowledge of the use of animations, especially since they can be easily and inexpensively shared digitally [9].

Limitations in the type of data, depth, and quality of reporting in studies on the design and implementation of public health interventions within published literature are well known [90]. Consequently, the pool of available literature for this review was reduced due to studies failing to provide access to the animation. Nevertheless, increasing interest in and application of health animations mean that the scientific evidence base will continue to expand.

Despite the aforementioned limitations, this review also has the following strengths. This is the first realist review of health animations designed for preventive health behavior promotion in the general population. We strictly adhered to the standards and guidance for conducting and reporting realist reviews [18,19,22], and as such, the review team engaged in regular discussion and reflection on the evidence identified. We actively and authentically included public and professional stakeholders throughout the review process, seeking to ensure that we involved international professional stakeholders from different sectors and public stakeholders from diverse ethnic and sociodemographic backgrounds. The resultant program theory extends the knowledge base by identifying and refining thinking around the questions of why, how, for whom, to what extent, and in which contexts health animations work as a digital form of health communication. The iterative, cyclical nature of realist reviews meant that searching for new evidence continued throughout the review process; therefore, noteworthy new evidence was captured and included in the synthesis. This new evidence reinforced initial findings shared with stakeholders and contributed to the currency of the review outcomes.

### Conclusions

Animations provide a compelling digital approach to support the promotion of preventive health behaviors. Their visual and entertaining nature, with reduced reliance on language, means that they can help reduce health literacy barriers in the target audience and address health inequities in populations. The findings of this realist review of health animations designed to promote preventive health behaviors, along with the program theory it has generated, provide novel insights into the specific contexts and mechanisms influencing evidence-based determinants of behavior and behavior change. It is critical that the contexts and mechanisms found to drive animation impacts in the evidence we reviewed, as well as the associated recommendations we provide, are considered by those designing and developing health animations in the future.

## Supplementary material

10.2196/79769 Multimedia Appendix 1 Ovid MEDLINE(R) search strategy.

10.2196/79769 Multimedia Appendix 2 Table of characteristics for peer-reviewed publications.

10.2196/79769 Multimedia Appendix 3 Table of characteristics for gray literature.

10.2196/79769 Multimedia Appendix 4 Table of constructs, contexts, and mechanisms.

## Abbreviations

CMOC: context mechanism outcome configuration
MeSH: Medical Subject Headings
RAMESES: Realist and Meta-Narrative Evidence Syntheses: Evolving Standards
