# Supplementary material for: Recognizing the Importance of Design, Content, and Delivery Features of Health Animations for Preventive Health Behaviors: Realist Review
Source: J Med Internet Res. 2026 Apr 23;28:e79769. doi: 10.2196/79769 (PMC13105399; doi:10.2196/79769)
Supplement: Multimedia Appendix 1 [file jmir-v28-e79769-s001.docx]

**Multimedia Appendix 1: Ovid MEDLINE(R) search strategy**

**1**  animat*.ti,ab,kf.
**2**  dynamic image*.ti,ab,kf.
**3**  dynamic picture*.ti,ab,kf.
**4**  moving picture*.ti,ab,kf.
**5**  moving image*.ti,ab,kf.
**6**  motion graphic*.ti,ab,kf.
**7**  explainer video*.ti,ab,kf.
**8**  cartoon*.ti,ab,kf.
**9**  or/1-8
**10**  exp Health Promotion/
**11**  exp Preventive Health Services/
**12**  Harm Reduction/
**13**  exp risk reduction behavior/
**14**  Health Communication/
**15**  (harm* or risk or reduc* or chang* or prevent* or health or intervention* or campaign* or promotion or communicat*).ti,ab,kf.
**16**  or/10-15
**17**  exp Health Behavior/
**18**  exp Obesity/
**19**  exp Weight Loss/
**20**  exp Exercise/
**21**  exp Diet/
**22**  exp Alcohol Drinking/
**23**  exp Smoking/
**24**  Sexual Health/
**25**  exp Sexually Transmitted Diseases/
**26**  exp Sleep/
**27**  exp Drug Misuse/
**28**  Oral Health/
**29**  exp Dental Care/
**30**  exp Air Pollution/
**31**  exp Hygiene/
**32**  exp Mass Screening/
**33**  exp Vaccination/
**34**  health check.ti,ab,kf.
**35**  exp Breast Feeding/
**36**  or/17-35
**37**  9 and 16 and 36
