# Supplementary material for: Recognizing the Importance of Design, Content, and Delivery Features of Health Animations for Preventive Health Behaviors: Realist Review
Source: J Med Internet Res. 2026 Apr 23;28:e79769. doi: 10.2196/79769 (PMC13105399; doi:10.2196/79769)
Supplement: Multimedia Appendix 2 [file jmir-v28-e79769-s002.docx]

**Multimedia Appendix 2: Table of characteristics for peer-reviewed publications**

| **Author/location/ design/ study title** | **Behavior/**  **participants/**  **delivery setting of animation** | **Outcome(s) measures** | **Animation characteristics**  Single animation or series/ entertainment category (see Table 1)/ sound/ language/ text/ length | **Associated CMOCs**  as defined/observed in this review^1^ |
| --- | --- | --- | --- | --- |
| Adam et al., 2021/ South Africa/ RCT/ Evaluation of a community-based mobile video breastfeeding intervention in Khayelitsha, South Africa: the Philani MOVIE cluster-randomized controlled trial [24] | Nutrition: Breast feeding/ adults (pregnant women)/ in-person, one-to-one with mentor mother, later telephone | Breastfeeding behaviors  Other baby feeding behaviors  Maternal knowledge | Series/ storytelling/ music/ narration AND dialogue between characters and/or interviews/ no text/ 2:00-5:00 min | Design CMOC:  1: Representation  2: Entertainment  3: Cognition  Content CMOC:  1: Emotion  2: Co-design  3: Function  Delivery CMOC:  1: Accessibility  2: Source |
| Adam et al., 2023/ South Africa/ RCT/ Effect of short, animated video storytelling on maternal knowledge and satisfaction in the perinatal period in South Africa: randomized controlled trial [25] | General health promotion: maternal and child health/ adults (expectant mothers)/ via WhatsApp | Knowledge of maternal and neonatal health  User satisfaction | Series/ storytelling/music AND background and/or illustrative sound/ narration AND dialogue between characters and/or interviews/ minimal text/ 1:33-4:22 min | Design CMOC:  1: Representation  2: Entertainment  3: Cognition  Content CMOC:  1: Emotion  2: Co-design  3: Function  Delivery CMOC:  1: Accessibility  2: Source  3: Exposure |
| Aeilts et al., 2021/ USA/ pre post study/ BRCAShare—Assessment of an animated digital message for intrafamilial communication of pathogenic variant positive test results: a feasibility study [26] | Screening: Genetic screening (breast cancer)/ adults/ online | Intentions (hypothetical) | Single/ storytelling elements/ music/ narration/ minimal text/ 2:00 min | Design CMOC:  2: Entertainment  3: Cognition  Content CMOC:  2: Co-design  3: Function  Delivery CMOC:  1: Accessibility  2: Source  3: Exposure |
| Aspiras et al., 2023/ USA/ randomised study/ Medical mistrust, culturally targeted message framing, and colorectal cancer screening among African Americans [27] | Screening: colorectal cancer/ adults (African Americans aged 50-75, never screened or off screening schedule)/ online | Medical mistrust  Receptivity to CRC screening (using TPB constructs)  Anticipatory racism | Series/entertainment elements/ music AND background and/or illustrative sound/ direct address/ minimal text/ 1:00-19:00 min | Design CMOC:  2: Entertainment  3: Cognition  Content CMOC:  1: Emotion  3: Function  Delivery CMOC:  1: Accessibility  2: Source  3: Exposure |
| Aspiras et al., 2023/ USA/ randomised study/ Culturally targeted message framing and colorectal cancer screening preferences among African Americans [28] | Screening: colorectal cancer/ adults (African Americans aged 50-75, never screened or off screening schedule)/ online | Preferred screening modality and desired thoroughness | Series/ entertainment elements/ music AND background and/or illustrative sound/ direct address/ minimal text/ 1:00-19:00 min | Design CMOC:  2: Entertainment  3: Cognition  Content CMOC:  1: Emotion  3: Function  Delivery CMOC:  1: Accessibility  2: Source  3: Exposure |
| Bayraktar Nahir et al., 2025/ Turkey/ cross-sectional study/ Primary school children's oral hygiene knowledge assessed with different educational methods: a cross-sectional study [29] | Oral health: oral hygiene/children (primary school students, aged 10-12)/ in-person group (classroom) | Knowledge | Single/ entertainment elements/ music/ direct address/ minimal text/ 1:00:17:00 min | Design CMOC:  2: Entertainment  3: Cognition  Content CMOC:  3: Function  Delivery CMOC:  1: Accessibility  2: Source  3: Exposure |
| Beleites et al., 2024/ USA/ RCT/ Evaluating the impact of short animated videos on COVID-19 vaccine hesitancy: an online randomized controlled trial [30] | Vaccination: COVID-19 hesitancy/ adults/ online | Knowledge, Behavioral intentions, participant engagement, self-perceptions | Series/ storytelling/music AND background and/or illustrative sound/ no language/ minimal text/ 4:00 min | Design CMOC:  2: Entertainment  3: Cognition  Content CMOC:  1: Emotion  3: Function  Delivery CMOC:  1: Accessibility  2: Source  3: Exposure |
| Bonner et al., 2024/ Australia/ RCT/ Using health literacy principles to improve understanding of evolving evidence in health emergencies: Optimization and evaluation of a COVID-19 vaccination risk-benefit calculator [31] | Vaccination: COVID-19/ adults/ online | Knowledge | Series/ entertainment elements/ music/ direct address/ heavy text/ 2:25 and 2:51 min | Design CMOC:  2: Entertainment  3: Cognition  Delivery CMOC:  1: Accessibility  2: Source  3: Exposure |
| Borzekowski et al., 2019/ Rwanda/ randomised study/ The impact of an educational media intervention to support children’s early learning in Rwanda [32] | General health promotion: range of general health behaviors/ children (aged 6-8)/ school site | Knowledge  Skills | Series/ storytelling/ music/ dialogue between characters and/or interviews/ no text/ 20:00-30:00 min | Design CMOC:  1: Representation  2: Entertainment  3: Cognition  Content CMOC:  3: Function  Delivery CMOC:  1: Accessibility  2: Source  3: Exposure |
| Brijnath et al., 2025/ Australia/ pre post study /Evaluating the effectiveness of a culturally adapted dementia prevention animation on ethnically diverse participants' knowledge of dementia prevention [33] | General health promotion: dementia prevention/ adults/ online and in-person | Knowledge | Single/entertainment elements/music/narration/no text/3-5 minutes (depending on language) | Design CMOC:  2: Entertainment  3: Cognition  Content CMOC:  2: Co-design  3: Function  Delivery CMOC:  1: Accessibility  2: Source |
| Burapasikarin et al., 2020/ Thailand/ RCT/ The effect of an educational video on long‐acting reversible contraception (LARC) utilization at 6–8 weeks postpartum period: a randomized controlled trial [34] | Sexual health: birth control/ adults (postpartum women aged 20+)/ postpartum ward of hospital | Percentage of LARC utilization at 6-8 weeks postpartum  Reasons for not using LARC | Single/ no storytelling or entertainment elements/ no sound/ narration/ heavy text/ 7:00 min | Design CMOC:  3: Cognition  Content CMOC:  3: Function  Delivery CMOC:  1: Accessibility  2: Source  3: Exposure |
| Crum et al., 2023/ New Zealand/ RCT/ Changing mindsets about side effects of the COVID-19 vaccination: a randomized controlled trial [35] | Vaccination: Covid-19/ adults (aged 18+, just received 2^nd^ Covid vaccination, spoke English)/ community vaccine sites on iPads | Mindset about symptoms  Side-effects (information evaluation; experiences; frequency; coping behaviors)  Future vaccine intentions | Single/ entertainment elements/ music AND background and/or illustrative sound/ direct address/ minimal text/ 3:42 min | Design CMOC:  2: Entertainment  3: Cognition  Content CMOC:  3: Function  Delivery CMOC:  1: Accessibility  2: Source  3: Exposure |
| Debenham et al., 2022/ Australia/ cross-sectional study/ Neuroscience literacy and substance use prevention: How well do young people understand their brain? [36] | Substance use: alcohol, cannabis, MDMA/ young adults (aged 16-24)/ online, Ministry of Health website | Neuroscience literacy levels  Evaluation of animations | Series/ entertainment elements/ background and/or illustrative sound/ narration/ minimal text/ 50 seconds | Design CMOC:  1: Representation  2: Entertainment  3: Cognition  Content CMOC:  3: Function  Delivery CMOC:  1: Accessibility  2: Source  3: Exposure |
| Debenham et al., 2025/ Australia/ mixed methods study/ Co-design of digital public health substance use resources: A collaboration between young people and experts [37] | Substance Use: e-cigarettes, antidepressants and alcohol use/ young adults/ online | Animation reception Perception of harm | Series/ entertainment elements/ background and/or illustrative sound/ narration/ minimal text/ 52-58 seconds | Design CMOC:  1: Representation  2: Entertainment  3: Cognition  Content CMOC:  2: Co-design  3: Function  Delivery CMOC:  1: Accessibility  2: Source  3: Exposure |
| Elling & de Vries, 2021/ Netherlands/ randomised study/ Influence of animation- versus text-  based delivery of a web-based computer-tailored smoking cessation intervention on user perceptions [15] | Substance use: smoking cessation/ adults (aged 18+)/ online | User experience  E-loyalty  Digital behavior change interventions engagement scale | Series/ entertainment elements/ music/ direct address/ minimal text/ 29 seconds –5:00 min | Design CMOC:  2: Entertainment  3: Cognition  Content CMOC:  3: Function  Delivery CMOC:  1: Accessibility  2: Source  3: Exposure |
| Ewald et al., 2023/ USA/ pre post study/ The path to good health: shifting the dialogue and promoting social ecological thinking [38] | General health promotion: social determinants of health/ adults/ setting information missing | 4-factor critical consciousness scale (passive adaptation; emotional engagement; cognitive awakening; intentions to act)  Political typology | Single/ storytelling elements/ music AND background and/or illustrative sound/ dialogue between characters and/or interviews AND direct address/ minimal text/ 4:02 | Design CMOC:  2: Entertainment  3: Cognition  Content CMOC:  3: Function  Delivery CMOC:  1: Accessibility  2: Source  3: Exposure |
| Favaretti et al., 2022/ UK/ RCT/ Participant engagement and reactance to a short, animated video about added sugars: web-based randomized controlled trial [39] | Nutrition: sugar reduction/ adults (aged 18-59, speak English, UK resident)/ online | Participant engagement  Trait reactance proneness | Single/ storytelling/ background and/or illustrative sound/ narration/ minimal text/ 3:42 min | Design CMOC:  2: Entertainment  3: Cognition  Content CMOC:  1: Emotion  3: Function  Delivery CMOC:  1: Accessibility  2: Source  3: Exposure |
| Gallegos-Jeffery et al., 2023/ USA/ mixed methods study/ Examining engagement and responses to a digital binge drinking campaign for young males in Florida [40] | Substance Use: alcohol consumption/ young adults/ online (social media) | Engagement  Animation reception | Series/ storytelling / music AND background and/or illustrative sound/ narration AND dialogue between characters or interviews/minimal text/ 1:06-3:40 min | Design CMOC:  1: Representation  2: Entertainment  3: Cognition  Content CMOC:  1: Emotion  Delivery CMOC:  1: Accessibility |
| Grigg et al., 2023/ Australia/ RCT/ A brief intervention for improving alcohol literacy and reducing harmful alcohol use by women attending a breast screening service: a randomised controlled trial [41] | Substance use: reduce alcohol consumption/ adults (women aged 40+, who report any alcohol use, not pregnant, not hearing impaired, read and understand English)/ at clinic, private viewing, iPad with earphones | Knowledge (alcohol as risk factor)  Change in alcohol literacy  Alcohol consumption | Single/ entertainment elements/ music/ narration/ minimal text/ 7:13 min | Design CMOC:  1: Representation  2: Entertainment  3: Cognition  Content CMOC:  1: Emotion  3: Function  Delivery CMOC:  1: Accessibility  2: Source  3: Exposure |
| Hachaturyan et al., 2021/ UK/ RCT/ Reactance to social authority in a sugar reduction informational video: web-based randomized controlled trial of 4013 participants [42] | Nutrition: sugar reduction/ adults (aged 18-59, speak English, UK resident)/ online | Differences in the antecedents to reactance (proneness to reactance, threat level of message), its elements (anger and negative cognition), and outcomes (source appraisal and attitude) | Single/ storytelling/ background and/or illustrative sound/ narration/ minimal text/ 3:42 min | Design CMOC:  2: Entertainment  3: Cognition  Content CMOC:  1: Emotion  3: Function  Delivery CMOC:  1: Accessibility  2: Source  3: Exposure |
| Haith-Cooper et al., 2020/ UK/ mixed methods study/ The co-development and feasibility-testing of an innovative digital animation intervention (DAISI) to reduce the risk of maternal sepsis in the postnatal period [43] | Communicable disease & hygiene behaviors: Postnatal sepsis/ adults (women)/ in-person | Understanding  Recall  Acceptability of animation | Single/entertainment elements/ no sound/ narration/ some text throughout/ 3:30 min | Design CMOC:  1: Representation  2: Entertainment  3: Cognition  Content CMOC:  2: Co-design  3: Function  Delivery CMOC:  1: Accessibility  2: Source |
| Hammarberg et al., 2024/ Australia/ cross-sectional study/ Development and evaluation of 4 short, animated videos for women in midlife promoting positive health behaviors: survey study [44] | General health promotion: Menopause health behaviors/ adult (women)/ online | Perspectives on videos (accessibility; acceptability; understanding; usability) | Series/ entertainment elements/music AND background and/or illustrative sound/ narration/ some text throughout/ 1:25-2:06 min | Design CMOC:  1: Representation  2: Entertainment  3: Cognition  Content CMOC:  3: Function  Delivery CMOC:  1: Accessibility  2: Source  3: Exposure |
| Harrison et al., 2023/ UK/ pre post study/ The effect of an educational animation on knowledge of testicular health and fertility of adolescents [45] | Sexual health: fertility and testicular health/ young adults (aged 13-14)/ in-person group | Knowledge Perceptions of animations | Series/ entertainment elements/ music/ narration/ some text throughout/ 1:33-2:13 min | Design CMOC:  1: Representation  2: Entertainment  3: Cognition  Content CMOC:  2: Co-design  3: Function  Delivery CMOC:  1: Accessibility  2: Source  3: Exposure |
| Jones et al., 2017/ New Zealand/ randomised study/ The impact of 3-D models versus animations on perceptions of osteoporosis and treatment motivation: a randomised trial [46] | General health promotion: osteoporosis awareness/ adults (female aged 50+, treatment naïve, understand English)/ group session at clinic | Perceptions  Beliefs  Motivations | Single/ no storytelling or entertainment elements/ no sound/ no language/ no text/ 15 seconds | Design CMOC:  3: Cognition  Delivery CMOC:  1: Accessibility |
| Kauffman et al., 2022/ Tanzania/ pre post study/ Emotions, strategies, and health: examining the impact of an educational program on Tanzanian preschool children [47] | General health promotion: range of general health behaviors / children (aged 3-9, low-income school, Arusha, Tanzania)/ after school, community viewing | Knowledge  Receptivity of media | Series/ storytelling/ music/ dialogue between characters and/or interviews/ no text/ 20:00-30:00 min | Design CMOC:  1: Representation  2: Entertainment  3: Cognition  Content CMOC:  3: Function  Delivery CMOC:  1: Accessibility  2: Source  3: Exposure |
| Lazic et al., 2021/ UK/ randomised study/ Social nudges for vaccination: How communicating herd behavior influences vaccination intentions [48] | Vaccination/ adults (aged 18-59, speak English, UK resident)/ online | Intentions | Single/ no storytelling or entertainment elements/ no sound/ no language/ some text throughout/ 27 seconds | Design CMOC:  3: Cognition  Content CMOC:  3: Function  Delivery CMOC:  1: Accessibility |
| Leiner et al., 2004/ USA/ RCT/ Patient communication: a multidisciplinary approach using animated cartoons [49] | Vaccination: polio/ parents/adults (caregivers of children receiving polio vaccine)/ clinic waiting room viewing | Knowledge acquisition | Single/ storytelling/ music AND background and/or illustrative sound/ narration/ minimal text/ 7:46 min | Design CMOC:  1: Representation  2: Entertainment  3: Cognition  Content CMOC:  1: Emotion  3: Function  Delivery CMOC:  1: Accessibility  3: Exposure |
| Lucas et al., 2021/ USA/ randomised study/ Effects of Culturally Targeted Message Framing on Colorectal Cancer  Screening Among African Americans [50] | Screening: colorectal cancer/ adults (African Americans aged 50-75, never screened or off screening schedule)/ online | Colorectal Cancer screening receptivity  FIT kit behavior  Anticipatory racism  Theory of Planned Behavior variables | Series/ Entertainment elements/ music AND background and/or illustrative sound/ direct address/ minimal text/ 1:00-19:00 min | Design CMOC:  2: Entertainment,  3: Cognition  Content CMOC:  1: Emotion  3: Function  Delivery CMOC:  1: Accessibility  2: Source  3: Exposure |
| Lucas et al., 2023/ USA/ randomised study/ Message framing for men? Gender moderated effects of culturally targeted message framing on colorectal cancer screening receptivity among African Americans [51] | Screening: colorectal cancer/ adults (African Americans aged 50-75, never screened or off screening schedule)/ online | Colorectal Cancer receptivity  Anticipatory racism  Potential for embarrassment | Series/ Entertainment elements/ music AND background and/or illustrative sound/ direct address/ minimal text/ 1:00-19:00 min | Design CMOC:  2: Entertainment  3: Cognition  Content CMOC:  1: Emotion  3: Function  Delivery CMOC:  1: Accessibility  2: Source  3: Exposure |
| Maisrikrod et al., 2023/ Australia, Thailand, Vietnam/ cross-sectional study/ Design and development of an internationally applicable educational video to increase community awareness in regions with high prevalence of melioidosis and diabetes [52] | Communicable disease & hygiene behaviors: Melioidosis prevention/ adults/ in-person group | Feedback on animation (cultural appropriateness; engaging, informative) | Single/ entertainment elements/ music/ narration/ some text throughout/ 4:03 min | Design CMOC:  2: Entertainment  3: Cognition  Content CMOC:  3: Function  Delivery CMOC:  1: Accessibility |
| Mansor et al., 2023/ Malaysia/ RCT/ Effectiveness of a theory-based digital animated video intervention to reduce intention and willingness to sext among diploma students: cluster randomized controlled trial [53] | Sexual health: sexting/ young adults (aged 18-24)/ online | Intentions  Knowledge | series/ entertainment elements/ music/ narration/heavy text/ 3:10-8:52 min | Design CMOC:  2: Entertainment  Content CMOC:  1: Emotion,  3: Function  Delivery CMOC:  1: Accessibility  2: Source  3: Exposure |
| Meppelink et al., 2015/ Netherlands/ randomised trial/ The effectiveness of health animations in audiences with different health literacy levels: an experimental study [14] | Screening: colon cancer/ adults (aged 55+, high or low education)/ online | Attitudes  Information recall  Intentions  Health literacy | Single/ no storytelling or entertainment elements/ no sound/ narration/ minimal text/ 3:15 min | Design CMOC:  3: Cognition  Content CMOC:  3: Function  Delivery CMOC:  1: Accessibility  2: Source  3: Exposure |
| Mohd Jaini et al., 2024/ Malaysia/ RCT/ Effectiveness of educational videos comparing single versus multiple topics: A cluster randomised controlled trial [54] | Oral health/ children (aged 5-6)/ in-person group (school room) | Plaque and gingival score  Oral health KAS (knowledge, attitude, practice) | Series/ storytelling/ music AND background and/or illustrative sound/ dialogue between characters or interviews/ minimal text/ 3:30-4:30 min | Design CMOC:  1: Representation  2: Entertainment  3: Cognition  Content CMOC:  3: Function  Delivery CMOC:  1: Accessibility  2: Source  3: Exposure |
| Molina-Salas et al., 2024/ Spain/ RCT/ Influence of the Olfatin Project on the reduction of pain related to intranasal influenza vaccination, as part of a school vaccination program [55] | Vaccination: Flu/ children (aged 3-4)/ in-person group (classroom) | Pain response after vaccine administration | Single/ storytelling/music AND background and/or illustrative sound/ narration AND direct address/no text / 2:30 min | Design CMOC:  2: Entertainment  3: Cognition  Content CMOC:  3: Function  Delivery CMOC:  1: Accessibility  2: Source  3: Exposure |
| O’Brien et al., 2022/ Guatemala/ pre post study/ A pre- and post-intervention study testing the effect of exposure to languageless animated images communicating COVID-19 preventive behaviors on behavioral intentions and beliefs of Guatemalan adults [56] | Communicable disease & hygiene behaviors: preventive behaviors, Covid-19/ adults (aged 18+, live in Guatemala, speak Spanish)/ online | Self-efficacy  Intentions  Outcome expectancy for hygiene behaviors | Series/ storytelling/ no sound/ no language/ no text/ 13-14 seconds | Design CMOC:  1: Representation,  2: Entertainment,  3: Cognition  Content CMOC:  2: Co-design  3: Function  Delivery CMOC:  1: Accessibility  2: Source  3: Exposure |
| Parsons et al., 2022/ UK/ pre post study/ Effectiveness of a digital intervention in increasing flu vaccination-related risk appraisal, intention to vaccinate and vaccination behavior among pregnant women [57] | Vaccination: flu/ adults (pregnant women aged 18+, resident in England, no flu vaccine that season)/ online | Risk appraisals  Intentions  Vaccination behaviors | Single/ storytelling/ music/ narration AND dialogue between characters and/or interviews/ heavy text/ 4:41 min | Design CMOC:  1: Representation  2: Entertainment  3: Cognition  Content CMOC:  1: Emotion  2: Co-design  3: Function  Delivery CMOC:  1: Accessibility  2: Source  3: Exposure |
| Ruparel et al., 2019/ UK/ randomized study/ Impact of a lung cancer screening information film on informed decision-making: a randomized trial [58] | Screening: lung cancer/ adults (smokers and former smokers (within 5 years of quitting) aged 60–75)/ in-person (with healthcare professional at hospital) | Knowledge  Decisional conflict  Screening participation  Acceptability of materials | Single/ storytelling elements/ music/ narration AND dialogue between characters and/or interviews/ minimal text/ 5:46 min | Design CMOC:  1: Representation  2: Entertainment  3: Cognition  Content CMOC:  1: Emotion  3: Function  Delivery CMOC:  1: Accessibility  2: Source  3: Exposure |
| Salmon et al., 2023/USA/ mixed methods study/ Let’s talk shots: personalized vaccine risk communication [59] | Vaccination: hesitancy/ adults (with vaccine concerns)/ online | Feedback on animations (clear; interesting; helpful; trustworthy) | Series/ entertainment elements/ music/ narration AND direct address/ minimal text/  < 2:00-6:00 min | Design CMOC:  2: Entertainment  3: Cognition  Content CMOC:  2: Co-design  3: Function  Delivery CMOC:  1: Accessibility  2: Source  3: Exposure |
| Schuh et al., 2023/ USA/ randomised study/ Evaluation of online videos to engage viewers and support decision-making for COVID-19 vaccination: how narratives and race/ ethnicity enhance viewer experiences [60] | Vaccination: COVID-19, vaccine hesitance/ adults/ online | Engagement  Feedback on animation | Series/ entertainment elements/ music/ narration AND direct address/ minimal text/  < 2:00-6:00 min | Design CMOC: 2: Entertainment 3: Cognition Content CMOC:  2: Co-design 3: Function Delivery CMOC: 1: Accessibility  2: Source  3: Exposure |
| Shukri et al., 2024/ Malaysia/ cross-sectional study/ Development and evaluation of educational video on healthy maternal diet to prevent child stunting [61] | Nutrition: Maternal and child health, stunting prevention/ adults (women aged 18-49 and nutrition experts)/ online | Evaluations of suitability  Understandability  Actionability | Series/ entertainment elements/ music/ narration/ some text throughout/ 3:33-5:27 min | Design CMOC:  2: Entertainment  3: Cognition  Content CMOC:  3. Function  Delivery CMOC:  1: Accessibility |
| Smith et al., 2024/ Australia/ RCT/ Randomised trial of information for older women about cessation of breast cancer screening invitations [62] | Screening: breast cancer/ adults (women aged 70-74)/ online | Knowledge  Attitudes  Intentions  Intentions to speak to GP  Perceived risk  Worry  Emotional response to letter received | Single/ entertainment elements/ music/ narration/ no text/ 1:50 min | Design CMOC:  1: Representation  2: Entertainment  Content CMOC:  3: Function  Delivery CMOC:  1: Accessibility  3: Exposure |
| Sumnall et al., 2024/ UK/ randomised study/ How to save a life: public awareness of a national mass media take home naloxone campaign, and effects of exposure to campaign components on overdose knowledge and responses [63] | Substance use: naloxone/adults/ online | Overdose knowledge Overdose actions (to be taken)  Readiness to intervene | Single/ entertainment elements/ music/ narration/ some text throughout/ 1:00 min | Design CMOC:  2: Entertainment  3: Cognition  Content CMOC:  1: Emotion  3: Function  Delivery CMOC:  1: Accessibility  2: Source  3: Exposure |
| Vandormael et al., 2021/ UK/ RCT/ Effect of a story-based, animated video to reduce added sugar consumption: a web-based randomized controlled trial [64] | Nutrition: sugar reduction/ adults (aged 18-59, speak English, UK resident)/ online | Behavioral intentions  Direct restoration of freedom | Single/ storytelling/ background and/or illustrative sound/ narration/ minimal text/ 3:42 min | Design CMOC:  2: Entertainment  3: Cognition  Content CMOC:  1: Emotion  3: Function  Delivery CMOC:  1: Accessibility  2: Source  3: Exposure |
| Vandormael et al., 2021/ USA, Mexico, UK, Germany, Spain/ RCT/ The effect of a wordless, animated, social media video intervention on COVID-19 prevention: online randomized controlled trial [16] | Communicable disease & hygiene behaviors: preventive behaviors Covid-19/ adults (aged 18–59)/ online | Intentions towards hygiene behaviors  Knowledge | Single/ storytelling/ background and/or illustrative sound/ no language/ no text/ 2:20 min | Design CMOC:  1: Representation  2: Entertainment  3: Cognition  Content CMOC:  1: Emotion  3: Function  Delivery CMOC:  1: Accessibility  2: Source  3: Exposure |
| Willis et al., 2018/ USA/ mixed methods study/ Developing a motion comic for HIV/STD prevention for young people ages 15-24, part 2: evaluation of a pilot intervention [65] | Sexual health: HIV/STD prevention/ young adults (aged 15-24, MSM and heterosexual males and females)/ in-person (during focus group) | HIV stigma and knowledge  Condom attitudes and knowledge  HIV testing attitudes  Behavioral intentions | Series/ storytelling/ background and/or illustrative sound/ dialogue between characters and/or interviews/ no text/ 38:00 min divided into 6 episodes | Design CMOC:  1: Representation  2: Entertainment  Content CMOC:  1: Emotion  2: Co-design  Delivery CMOC:  1: Accessibility  2: Source  3: Exposure |
| Witus & Larson, 2022/ USA/ RCT/ A randomized controlled trial of a video intervention shows evidence of increasing COVID-19 vaccination intention [66] | Vaccination: Covid-19/ adults (in USA, not vaccinated for Covid-19)/ online | Intentions  Intentions against political ideology | Single/ entertainment elements/ music/ narration/ some text throughout/ 8:00 min | Design CMOC:  2: Entertainment  3: Cognition  Content CMOC:  3: Function  Delivery CMOC:  1: Accessibility  2: Source |
| Zhu et al., 2022/ Canada/ RCT/ The Efficacy of a brief, altruism-eliciting video intervention in enhancing COVID-19 vaccination intentions among a population-based sample of younger adults: randomized controlled trial [67] | Vaccination: Covid-19/ adults (aged 20-39, Canada resident, not vaccinated for Covid-19)/ online | Intentions | Single/ storytelling elements/ background and/or illustrative sound/ narration/ minimal text/ 3:00 min | Design CMOC:  2: Entertainment  3: Cognition  Content CMOC:  1: Emotion  3: Function  Delivery CMOC:  1: Accessibility |

^1^If a CMOC is not listed as being associated with a particular animation, it does not necessarily mean that the animation does not represent or reflect that particular context/mechanism, but that sufficient information to confirm its association could not be located**.**
