# Supplementary material for: Recognizing the Importance of Design, Content, and Delivery Features of Health Animations for Preventive Health Behaviors: Realist Review
Source: J Med Internet Res. 2026 Apr 23;28:e79769. doi: 10.2196/79769 (PMC13105399; doi:10.2196/79769)
Supplement: Multimedia Appendix 3 [file jmir-v28-e79769-s003.docx]

**Multimedia Appendix 3: Table of characteristics for gray literature**

| **Producer/ name of animation/ year launched/ URL** | **Focus/ target audience/ location** | **Impact and further information** (information taken from producer/ NGO/animator websites when available) | **Animation characteristics**  Single animation or series/ entertainment category (see Table 1)/ sound/ language/ text/ length | **Associated CMOCs**  as defined/observed in this review^1^ |
| --- | --- | --- | --- | --- |
| El Laboratorio/ COVIDLatino/ 2020/ https://covidlatino.org/ [68] | Communicable disease & hygiene behaviors: Covid-19/Latinos/ USA | Over 3 million views Unpublished data show acceptability and increased knowledge  Well received by community | Series/ storytelling/ music AND background and/or illustrative sound/ dialogue between characters and/or interviews AND direct address/ minimal text/ 1:00-3:00 min | Design CMOC:  1: Representation  2: Entertainment  3: Cognition  Content CMOC:  1: Emotion  2: Co-design  3: Function  Delivery CMOC:  2: Source  3: Exposure |
| Global Health Media/ The story of cholera/ 2010/ https://globalhealthmedia.org/our-projects/cholera-series/ [69] | Communicable disease & hygiene behaviors: Cholera/ general population/ Haiti (initially), now global, the film is used in diverse settings such as refugee camps in Lebanon and state-wide training programs in India | >135 million views, 53 languages  Widely used for teaching sanitation and hygiene  Senior staff from UNICEF and other humanitarian organizations have praised the film’s ability to empower local populations to improve their hygiene and protect themselves from cholera | Single/ storytelling/ music AND background and/or illustrative sound/ narration/ no text/ 4:28 min | Design CMOC:  1: Representation  2: Entertainment  3: Cognition  Content CMOC:  1: Emotion  3: Function  Delivery CMOC:  1: Accessibility |
| Global Health Media/ The story of coronavirus/ 2020/ https://globalhealthmedia.org/our-projects/coronavirus-series/ [70] | Communicable disease & hygiene behaviors: Covid-19/ general population/ global | As of July 2021, 45 languages represented and over 25 million views  Featured as a case study by the WHO  Won ≥39 awards and widely shared across social media platforms | Single/ storytelling/ music AND background and/or illustrative sound/ narration/ no text/ 3:58 min | Design CMOC:  1: Representation  2: Entertainment  3: Cognition  Content CMOC:  1: Emotion  3: Function  Delivery CMOC:  1: Accessibility |
| Global Health Media/ The story of ebola/ 2014/ https://globalhealthmedia.org/our-projects/ebola-series/ [71] | Communicable disease & hygiene behaviors: Ebola/ general population/ Liberia, Sierra Leone, Guinea, Congo, DRC, shown to community leaders, traditional chiefs, and villages with laptops and small projectors, also shown in Ebola treatment centres | Over 60 million views on YouTube  83+million other views 4 languages represented  Used extensively by aid organizations | Single/ storytelling/ music AND background and/or illustrative sound/ narration/ no text/ 7:24 min | Design CMOC:  1: Representation  2: Entertainment  3: Cognition  Content CMOC:  1: Emotion  3: Function  Delivery CMOC:  1: Accessibility |
| UNESCO India/ Health and well-being of adolescents/ 2020/ https://www.unesco.org/en/articles/new-animation-videos-promote-health-and-well-being-adolescents [72] | General health promotion: range of general health behaviors/ children and young adults/ India | Part of school health program within the National Education Policy 2020, 5 of 11 modules are focused on health topics | Series/ storytelling/ music AND background and/or illustrative sound/ dialogue between characters and/or interviews/ no text/ 4:00-7:00 min | Design CMOC:  1: Representation  2: Entertainment  3: Cognition  Content CMOC:  3: Function |
| UNICEF/ Meena/ 1993/ https://www.unicef.org/bangladesh/en/meena-and-unicef [73] | General health promotion: range of general health behaviors/ children and young girls/ South Asia (initially), now broadcast in India, Bangladesh, Pakistan, Sri Lanka, Nepal, Bhutan, Laos, Cambodia, Vietnam | Meena is recognized by ~97% urban, 81% rural children in Bangladesh (the first country to broadcast)  In its development UNICEF consulted with over 10,000 children to design character's life, clothes, etc | Series/ storytelling/ music AND background and/or illustrative sound/ dialogue between characters and/or interviews AND direct address/ no text/ 13:00-20:00 min | Design CMOC:  1: Representation  2: Entertainment  3: Cognition  Content CMOC:  2: Co-design  3: Function  Delivery CMOC:  1: Accessibility  2: Source |
| UNICEF Ghana/ Cholera animation/ 2016/ https://youtu.be/G954-nmMAzg?si=jN1n8Pv5Jmi_tgTB [74] | Communicable disease & hygiene behaviors: Cholera/ general population/ Ghana | >500 thousand views on YouTube | Single/ storytelling/ music AND background and/or illustrative sound/ dialogue between characters and/or interviews/ no text/ 5:40 min | Design CMOC:  1: Representation  2: Entertainment  Content CMOC:  1: Emotion  3: Function |
| WHO Eastern Mediterranean Region/ Cholera – you can fight it too/ 2018/ https://youtu.be/8-sqticNg5o?si=khkPX7rYLAKieCWv [75] | Communicable disease & hygiene behaviors: Cholera/ general population/ Afghanistan, Bahrain, Djibouti, Egypt, Iran, Iraq, Jordan, Kuwait, Lebanon, Libya, Morocco, Oman, Pakistan, Qatar, Saudi Arabia, Somalia, Sudan, Syria, Tunisia, United Arab Emirates, Yemen |  | Single, but characters used in other animations not included in this review/ storytelling/ music AND background and/or illustrative sound/ narration AND dialogue between characters and/or interviews/ minimal text/ 3:20 min | Design CMOC:  1: Entertainment  3: Cognition  Content CMOC:  1: Emotion  3: Function |
| PAHO, Canadian government/ Intercultural dialogue and the mojojoy fable/ 2024/ https://youtu.be/FuytMCGNHGk?si=xANPnm8GvsmrOGZM [76] | Communicable disease & hygiene behaviors: trachoma/ general population/ border communities between Colombia and Brazil | Well received by target audience | Single/ storytelling, full/ music AND background and/or illustrative sound/ narration AND dialogue between characters and/or interviews/ no text/ 6:10 min | Design CMOC:  1: Representation  2: Entertainment  3: Cognition  Content CMOC:  2: Co-design  3: Function |

^1^If a CMOC is not listed as being associated with a particular animation, it does not necessarily mean that the animation does not represent or reflect that particular context/mechanism, but that sufficient information to confirm its association could not be located
