# Supplementary material for: Recognizing the Importance of Design, Content, and Delivery Features of Health Animations for Preventive Health Behaviors: Realist Review
Source: J Med Internet Res. 2026 Apr 23;28:e79769. doi: 10.2196/79769 (PMC13105399; doi:10.2196/79769)
Supplement: Multimedia Appendix 4 [file jmir-v28-e79769-s004.docx]

**Multimedia Appendix 4: Table of constructs, contexts, and mechanisms**

| **Construct** | **Context(s)** | **Mechanism(s)** |
| --- | --- | --- |
| Design | Representation   - Representation - Audience or problem | Identification  Attention  Thinking |
|  | Entertainment   - Storytelling - Entertainment - Storytelling or entertainment elements | Transportation  Attention  Vicarious experiences |
|  | Cognition   - Design features that facilitate efficient processing (i.e. use of metaphor/ symbols, length, sound, language, text) | Cognitive processes (i.e. memory, recall, thinking, learning, attention, processing) |
| Content | Emotion   - Emotionally evocative presentation of content | Elicitation of powerful feelings,  thoughts, emotions |
|  | Co-design   - Content that is co-designed with members of the target audience | Increased perception of personal relevance  Acceptability needs met |
|  | Function   - Challenge presented with solution | Building self-efficacy  Understanding  Know-how  Modelling  Motivation  Learning |
| Delivery | Accessibility   - Accessibility (i.e. in-person, online, group vs. private, appropriate literacy level) | Increased engagement  Understanding |
|  | Source   - Credibly perceived source (i.e. producer, platform, person, setting) | Building Trust  Engagement  Belief  Attention  Memory and recall |
|  | Exposure   - Mode, channel, intensity (i.e. auditory/visual channels, pathways, single animation vs. series) | Reduced cognitive load  Optimized cognitive processing |
